# The antiparasitic drug niclosamide inhibits dengue virus infection by interfering with endosomal acidification independent of mTOR

**DOI:** 10.1371/journal.pntd.0006715

**Published:** 2018-08-20

**Authors:** Jo-Chi Kao, Wei-Chun HuangFu, Tsung-Ting Tsai, Min-Ru Ho, Ming-Kai Jhan, Ting-Jing Shen, Po-Chun Tseng, Yung-Ting Wang, Chiou-Feng Lin

**Affiliations:** 1 Department of Microbiology and Immunology, School of Medicine, College of Medicine, Taipei Medical University, Taipei, Taiwan; 2 Graduate Institute of Medical Sciences, College of Medicine, Taipei Medical University, Taipei, Taiwan; 3 Ph.D. Program for Cancer Molecular Biology and Drug Discovery, College of Medical Science and Technology, Taipei Medical University, Taipei, Taiwan; 4 Graduate Institute of Cancer Biology and Drug Discovery, College of Medical Science and Technology, Taipei Medical University, Taipei, Taiwan; University of Texas Medical Branch, UNITED STATES

## Abstract

**Background:**

The antiparasitic agent niclosamide has been demonstrated to inhibit the arthropod-borne Zika virus. Here, we investigated the antiviral capacity of niclosamide against dengue virus (DENV) serotype 2 infection *in vitro* and *in vivo*.

**Principle finding:**

Niclosamide effectively retarded DENV-induced infection *in vitro* in human adenocarcinoma cells (A549), mouse neuroblastoma cells (Neuro-2a), and baby hamster kidney fibroblasts (BHK-21). Treatment with niclosamide did not retard the endocytosis of DENV while niclosamide was unable to enhance the antiviral type I interferon response. Furthermore, niclosamide did not cause a direct effect on viral replicon-based expression. Niclosamide has been reported to competitively inhibit the mTOR (mammalian target of rapamycin), STAT3 (signal transducer and activator of transcription 3), and NF-κB (nuclear factor kappa-light-chain-enhancer of activated B cells) signaling pathways; however, selective inhibitors of those pathways did not reduce DENV infection. Similar to the vacuolar-type H^+^-ATPase inhibitor bafilomycin A1, both niclosamide and other protonophores, such as CCCP (carbonyl cyanide m-chlorophenyl hydrazone), and FCCP (carbonyl cyanide-p-trifluoromethoxyphenylhydrazone), effectively reduced endosomal acidification and viral dsRNA replication. Co-administration of a single dose of niclosamide partially decreased viral replication, viral encephalitis, and mortality in DENV-infected ICR suckling mice.

**Significance:**

These results demonstrate that niclosamide diminishes viral infection by hindering endosomal acidification.

## Author summary

Dengue and severe dengue cause global health concerns annually. Without antiviral drugs, supportive care is the only treatment option for patients with DENV infection. A current vaccine has been approved for protection against DENV infection; however, the potential risks and challenges associated with the immunopathogenesis of DENV remain unresolved. For anti-dengue therapy, the repurposing of drugs with antimicrobial and anticancer properties is a possible pharmacological strategy. In this study, we evaluated the potential antiviral effects of the antiparasitic drug niclosamide, considering its current pharmacological efficacy against arthropod-borne Zika virus infection. Using *in vitro* and *in vivo* models of DENV infection, we demonstrated that one of the therapeutic effects of niclosamide is to significantly target endosomal acidification. Following safety screening, repurposing niclosamide treatment may facilitate the development of anti-dengue drugs in the near future.

## Introduction

Dengue virus (DENV), which is transmitted by the bite of mosquitoes of the *Aedes* genus, causes approximately 390 million infections annually [1]. DENV belongs to the genus *Flavivirus* of the family *Flaviviridae* with a single-stranded, positive sense RNA genome approximately 11 kb in length [2]. The genome of DENV contains a single open reading frame encoding a polyprotein precursor, which is further cleaved into three structural proteins (capsid (C), premembrane (prM), and envelope (E) proteins) and seven non-structural proteins (NS1, NS2A, NS2B, NS3, NS4A, NS4B, and NS5), which have roles in the pathogen-host interaction and pathogenesis [3]. Patients with DENV infection are usually asymptomatic. However, 3 to 14 days after the infective mosquito bite, some patients exhibit extreme symptoms, including headache, vomiting, fever, rash, myalgia, and retro-orbital pain. Moreover, some patients further progress to life-threatening severe DENV infection, which is characterized by CNS impairment, multiple organ failure, plasma leakage and severe bleeding (dengue hemorrhagic fever and dengue shock syndrome) [4]. To date, there is no effective antiviral drug available for blocking DENV infection.

In addition to its function as an anthelmintic drug, niclosamide has been widely reported to confer broad antiviral activity [5]. Zika virus, a member of the *Flavivirus* genus, has been reported to be inhibited by niclosamide treatment through an unknown therapeutic mechanism [6]. Niclosamide likely inhibits Zika and DENV infection through an undefined targeting of flavivirus NS2B-NS3 protease [7]. Repurposing the application of niclosamide for anti-flavivirus therapy is a proposed strategy. However, niclosamide also confers multifaceted blocking effects on different virus infection as well as tumorigenesis. In Epstein-Barr virus infection, niclosamide suppresses viral lytic replication by inhibiting mTOR (mammalian target of rapamycin) activation [8]. Additionally, niclosamide exhibits anticancer activity by blocking the mTOR, STAT3 (transducer and activator of transcription 3), and NF-κB (nuclear factor kappa-light-chain-enhancer of activated B cells) signaling pathways [9, 10]. Although these molecules have been reported to be involved in viral infection [11], and targeting mTOR may facilitate DENV replication through autophagy induction [12, 13], the potent antiviral effects of niclosamide against DENV infection warrant further investigation.

The infectious processes of DENV include viral adherence (receptor-mediated), entry (endocytosis-mediated), fusion and uncoating from endosomes following endosomal acidification, RNA release and replication, protein translation, virion assembly, and release [14]. Targeting these processes is a proposed antiviral strategy. Following endocytosis, endosomal acidification leads to the fusion of the viral envelope protein with the host membrane, facilitating the release of the viral genome [15]. Vacuolar-type H^+^-ATPase (V-ATPase), the proton-pumping enzyme that generates the low intra-vacuolar pH, is required for DENV endocytosis and infection *in vitro* [16]. Genetically and pharmacologically targeting V-ATPase effectively retard DENV infection *in vivo* [17, 18]. Jurgeit *et al*. demonstrated that niclosamide acts as a proton carrier which blocks endosomal acidification to inhibit human rhinovirus and influenza virus infection [19]. Using our previous *in vitro* and *in vivo* models of DENV infection [17, 20], we investigated the possible antiviral effects and molecular actions of niclosamide on blocking DENV infection.

## Materials and methods

### Cells, virus strains, and reagents

Murine Neuro-2a cells (ATCC, CCL131), human A549 (ATCC, CCL185), and baby hamster kidney (BHK)-21 cells (ATCC, CCL10) were cultured in Dulbecco’s modified Eagle's medium (DMEM; Invitrogen Life Technologies, Rockville, MD). *Aedes albopictus* C6/36 cells (ATCC, CRL1660) were grown on plastic in RPMI medium 1640 (RPMI; Invitrogen Life Technologies). All culture media were supplemented with 10% heat-inactivated fetal bovine serum (FBS; Invitrogen Life Technologies), 50 U/mL penicillin and 50 μg/mL streptomycin. DENV2 PL046, a Taiwanese human isolate obtained from the Centers for Disease Control in Taiwan, was propagated in C6/36 cells. Viral titers were quantified by plaque assay using the BHK-21 cells accordingly [17, 20]. The following reagents and antibodies were used in these studies: niclosamide, the mTOR inhibitor rapamycin, the STAT3 inhibitor Cucurbitacin I, the NF-κB inhibitor BAY 11–7082, the V-ATPase inhibitor bafilomycin A1, protonophores carbonyl cyanide m-chlorophenyl hydrazone (CCCP) and carbonyl cyanide-p-trifluoromethoxyphenylhydrazone (FCCP), Hoechst 33258, dimethyl sulfoxide (DMSO), acridine orange, and mouse mAb specific for β-actin (Sigma-Aldrich, St. Louis, MO); antibodies against Akt Ser473, Akt, p70S6K Thr389, p70S6K, ERK1/2 Thr202/Tyr204, and ERK1/2 (Cell Signaling Technology, Beverly, MA); antibodies against dsRNA, DENV NS1, NS3, capsid, and E (GeneTex, San Antonio, TX); polyclonal anti-rabbit Atg8 (LC3) I/II (MBL international, Nagoya, Japan); goat anti-rabbit IgG conjugated with HRP (Chemicon International, Temecula, CA); rabbit anti-mouse IgG conjugated with HRP (Abcam, Cambridge, MA); and Alexa Fluor 488-conjugated goat anti-mouse (Invitrogen, Carlsbad, CA).

### Animals

The animal experiments were performed according to the guidelines of the Animal Protection Act of Taiwan. Protocols according to guidelines established by the Ministry of Science and Technology, Taiwan were approved by the Laboratory Animal Care and Use Committee of National Cheng Kung University (Approval number IACUC #104062). Seven-day-old ICR suckling mice were inoculated intracerebrally with 2.5 × 10^5^ plaque-forming units (pfu) and intraperitoneally with 7.5 × 10^5^ pfu of DENV2 (PL046), which was combined with or without niclosamide (2 or 5 mg/kg) treatment. On day 9 post-infection, brain tissue was harvested for the protein assay. Body weight and disease scoring were carried out according to our previous studies [17, 20].

### DENV infection

Cells were resuspended at a concentration of 7 × 10^4^ or 1 × 10^5^ cells/mL in the appropriate medium with DENV (MOI = 1) and incubated for 2 h at 37°C. The cells were then washed once with culture medium and incubated for the indicated times. The presence of viral supernatants was evaluated using plaque assays.

### Plaque assay

BHK-21 cells were plated onto 12-well plates (7 × 10^4^ cells/well). After adsorption with a serially diluted virus solution for 2 h, the solution was replaced with fresh DMEM containing 4% FBS and 0.5% methyl cellulose (Sigma-Aldrich). Five days post-infection, the medium was removed, and the cells were fixed and stained with crystal violet solution containing 1% crystal violet, 0.64% NaCl, and 2% formalin.

### Cytotoxicity

Cell cytotoxicity was assessed using Cytotoxicity Detection kit assays (Roche Diagnostics, Lewes, UK) according to the manufacturer’s instructions.

### Western blotting

Total cell lysates were extracted with a buffer containing 1% Triton X-100, 50 mM Tris (pH 7.5), 10 mM EDTA, 0.02% NaN_3_, and a protease inhibitor mixture (Roche Applied Science, Indianapolis, IN). Proteins were separated using SDS- polyacrylamide gel electrophoresis and transferred to a polyvinylidene difluoride membrane (Millipore Corporation, Billerica, MA). After blocking, blots were probed with the indicated antibodies and developed using enhanced chemiluminescence (Pierce, Rockford, IL). Following densitometry-based quantification and analysis using ImageJ software (http://rsbweb.nih.gov/ij/), the relative density of each identified protein was calculated.

### Reporter assay

BHK-21 cells harboring a luciferase-expressing DENV replicon (BHK-D2-Fluc-SGR-Neo-1) were generated and maintained according to previous studies [17].

### Fluorescent DENV

Fluorescent DENV was prepared by labeling with Alexa Fluor 594 succinimidyl ester (AF594SE, Molecular Probes, Invitrogen) as referred to the previous studies [21]. The labeled viruses were purified using Amicon Ultra-15 PLTK Ultracel-PL Membrane (30 kDa) centrifugal filter units (Millipore) to remove excess dye. Cells were washed twice after an inoculation (MOI = 1) with cells for 2 h at 37°C. Cells were visualized under a laser-scanning confocal microscope (Leica TCS SP5 confocal microscope (Leica Microsystems, Mannheim, Germany) and were analyzed using FACSCanto II Flow cytometer (BD Biosciences, Franklin Lakes, NJ). The three-dimensional images reconstructed from a series of confocal images, along with the z-axis of the cells and the analysis of z-stacks, were reconstructed using the Leica Confocal Software.

### Immunostaining

Cells were fixed with 4% paraformaldehyde, permeabilized with 0.5% Triton X-100, and washed twice with ice-cold phosphate-buffered saline. Cells were first probed with anti-dsRNA antibodies [22] and then probed with Alexa 488-conjugated goat anti-mouse IgG. 4',6-diamidino-2-phenylindole (DAPI, 5 μg/mL) was used for nuclear staining. Cells were visualized under a fluorescence microscope (EVOS FL cell imaging system, Thermo Fisher Scientific, Waltham, MA) or analyzed using flow cytometry (Attune Nxt). The mean fluorescence intensity (MFI) of the dsRNA was analyzed with ImageJ software.

### ELISA

The concentration of IFN-β in the cell-conditioned culture medium was determined using ELISA kits (PBL Assay Science, Piscataway, NJ) according to the manufacturer’s instructions.

### AO staining

Cells were treated with 5 ng/mL acridine orange (AO; Sigma-Aldrich) in a serum-free culture medium for 30 min at 37°C. After being washed with Hank's balanced salt solution twice, cells were visualized under a fluorescence microscope (EVOS).

### Statistical analysis

Data obtained from three independent experiments are presented as the mean ± standard deviation (SD). Two sets of data were analyzed by an unpaired Student’s *t* test. Three or more sets of data were analyzed by one-way ANOVA with Tukey’s multiple-comparison test. Statistical significance was set at *P* < 0.05.

## Results

### Niclosamide treatment decreases DENV infection *in vitro*

**Fig 1:**
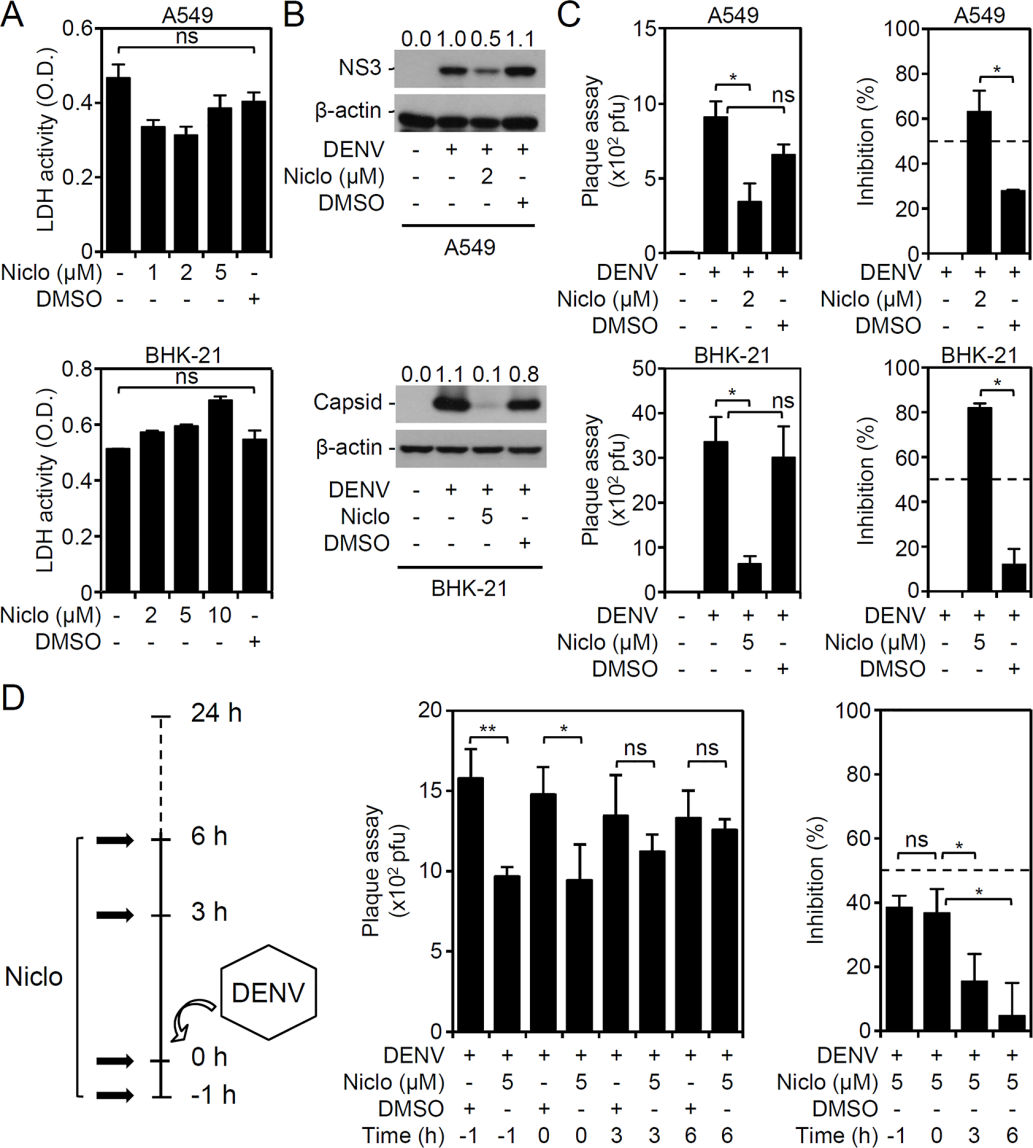
Niclosamide treatment reduces viral protein expression and viral release in DENV serotype 2 PL046 (DENV2)-infected cells. (A) LDH assays showing cytotoxicity in niclosamide (Niclo)-treated A549 (48 h) and BHK-21 (24 h) cells at various concentrations. (B) Representative Western blots showing viral NS3 and capsid protein expression. β-actin served as the internal control. The relative ratios of the measured proteins compared to β-actin are also shown. (C) Plaque assays showing viral release in DENV2 (MOI = 1)-infected A549 (48 h) and BHK-21 (24 h) cells in the presence of niclosamide (Niclo). (D) Plaque assays showing the production of infectious particles for a kinetic treatment (pre-, co-, and post-treatment) of niclosamide (Niclo) in DENV2 (MOI = 1)-infected BHK-21 (24 h) cells. Virus particles are shown as the desired pfu amount for infection and as calculated as the percentage (%) of inhibition. DMSO was used as a solvent control. The quantitative data are depicted as the mean ± SD of three independent experiments. * *P <* 0.05 and ** *P <* 0.01. ns, not significant.

Niclosamide confers potential anti-flavivirus activity against Zika virus infection by targeting unknown factors [6]. It is hypothesized that treatment with niclosamide inhibits not only Zika virus but also the flavivirus DENV. To verify the antiviral effects of niclosamide against DENV infection, an *in vitro* cell model of DENV infection was examined for viral protein expression and viral release [17]. The release of LDH was measured to monitor cytotoxicity in A549 and BHK-21 cells, and it was found that treatment with niclosamide at all sub-lethal doses caused minor cytotoxic effects on these cells **(Fig 1A)**. Evaluation of 50% cytotoxic concentration (CC50) on niclosamide-treated BHK-21 cells by using LDH assay showed that the value was less than 10 μM **(S1 Fig)**. Further cytotoxic response as assessed by rhodamine 123-based staining for monitoring mitochondrial membrane potential loss was carried out to exclude the lethal dose of niclosamide used in this study **(S2 Fig)**. Niclosamide effectively blocked viral protein expression **(Fig 1B)** and significantly (*P <* 0.05) retarded viral release **(Fig 1C)**. Additionally, niclosamide showed a value of half maximal effective concentration (EC50) near 10 μM **(S3 Fig)**. Under usage with niclosamide at 5 μM, pre- and co-administration but not post-treatment significantly (*P <* 0.05) inhibited DENV replication **(Fig 1D)**. These results confirm the antiviral effect of niclosamide treatment against DENV infection *in vitro*.

### Blocking DENV infection by niclosamide does not affect the endocytosis of DENV or type I IFN response

**Fig 2:**
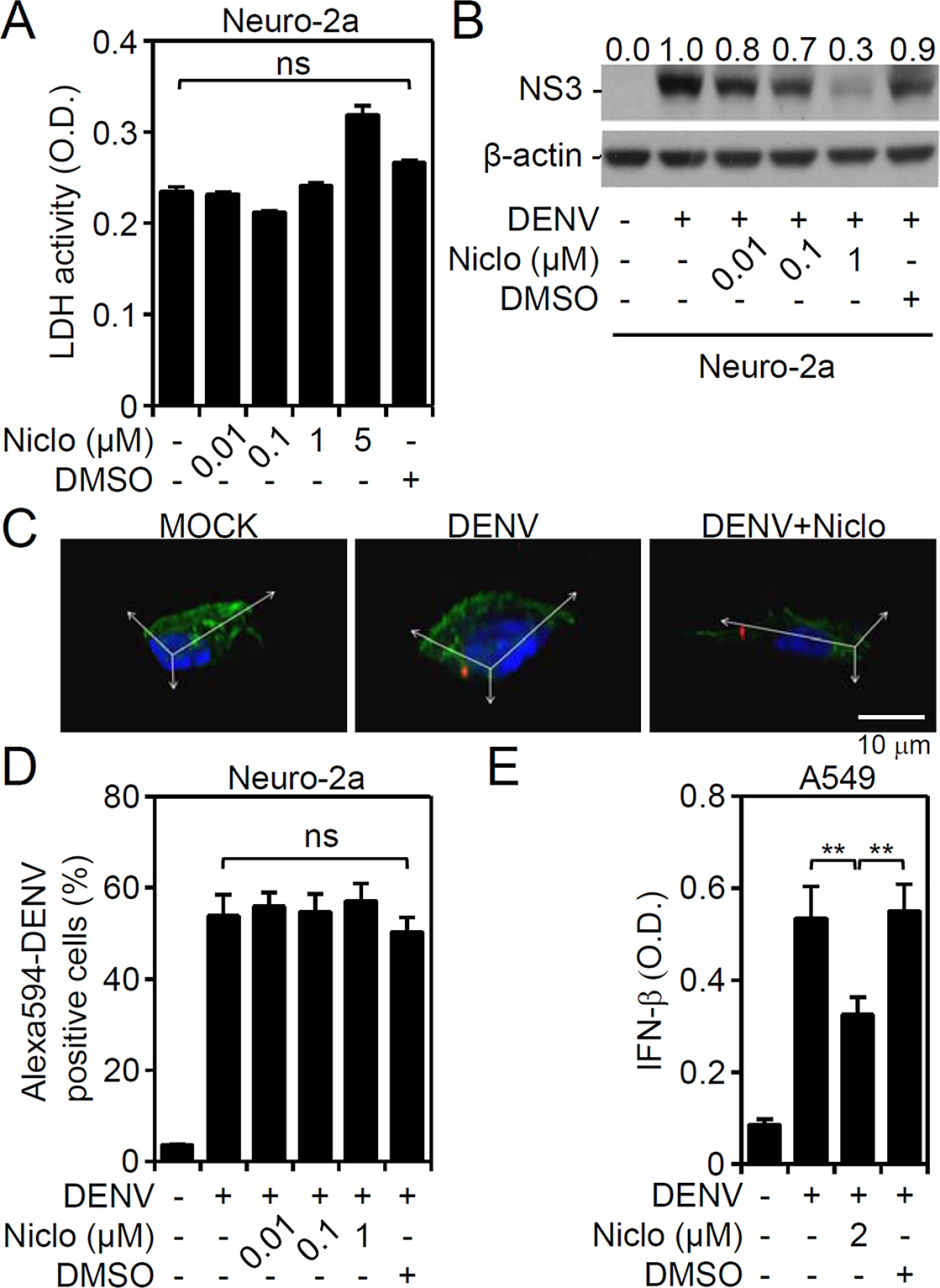
Niclosamide treatment neither hinders the endocytosis of DENV nor enhances IFN-β production. **(**A) LDH assay showing cytotoxicity in niclosamide (Niclo)-treated Neuro-2a cells for 48 h with various concentrations. (B) Representative Western blot showing viral NS3 protein expression in DENV2 (MOI = 1)-infected Neuro-2a cells for 48 h with or without niclosamide (Niclo) pretreatment. β-actin served as the internal control. The relative ratios of the measured proteins compared to β-actin are also shown. Confocal microscopy (C) and flow cytometry (D) were used to measure the positive Neuro-2a cells carrying Alexa-594 labeled (*red*) DENV2 (MOI = 1) 2 h post-infection in the presence of niclosamide (Niclo). E: ELISA analysis showing IFN-β production in DENV2 (MOI = 1)-infected A549 cells for 48 h with or without niclosamide (Niclo) pretreatment. DMSO was used as a solvent control. The quantitative data are depicted as the mean ± SD of three independent experiments. ** *P <* 0.01. ns, not significant.

Next, to investigate the possible antiviral actions of niclosamide, the cellular responses and infectious processes during DENV infection were explored. We previously demonstrated the infectivity of DENV in Neuro-2a cells [17]. The cytotoxic effects of niclosamide at sub-lethal doses were monitored **(Fig 2A)**. The Western blot results showing the inhibition of DENV NS3 protein expression in the presence of niclosamide treatment confirmed the antiviral effect of niclosamide in DENV-infected Neuro-2a cells **(Fig 2B)**. To demonstrate the infection efficacy in Neuro-2a cells, we performed fluorescent DENV staining followed by confocal microscopic observation **(Fig 2C)** as well as flow cytometric analysis **(Fig 2D)**. The results showed viral endocytosis at 2 h post-inoculation, which was not retarded by niclosamide treatment. Our findings exclude the possibility of niclosamide blocking viral endocytosis at the early stage of DENV infection.

To identify the target of niclosamide underlying its antiviral capacity, the type I IFN response was monitored regarding its potent antiviral effect in response to DENV infection. In DENV-infected A549 cells, as quantified by ELISA, IFN-β production was significantly (*P <* 0.01) increased **(Fig 2E)**. We next examined whether niclosamide treatment enhances IFN-β production to reduce DENV infection. However, the results revealed that niclosamide significantly (*P <* 0.01) decreased IFN-β production **(Fig 2E)**, probably following an early blockade on viral infection. These data imply a role of niclosamide independent of facilitating the antiviral type I IFN response.

### No effects on viral genome replication in niclosamide-treated DENV replicon cells

**Fig 3:**
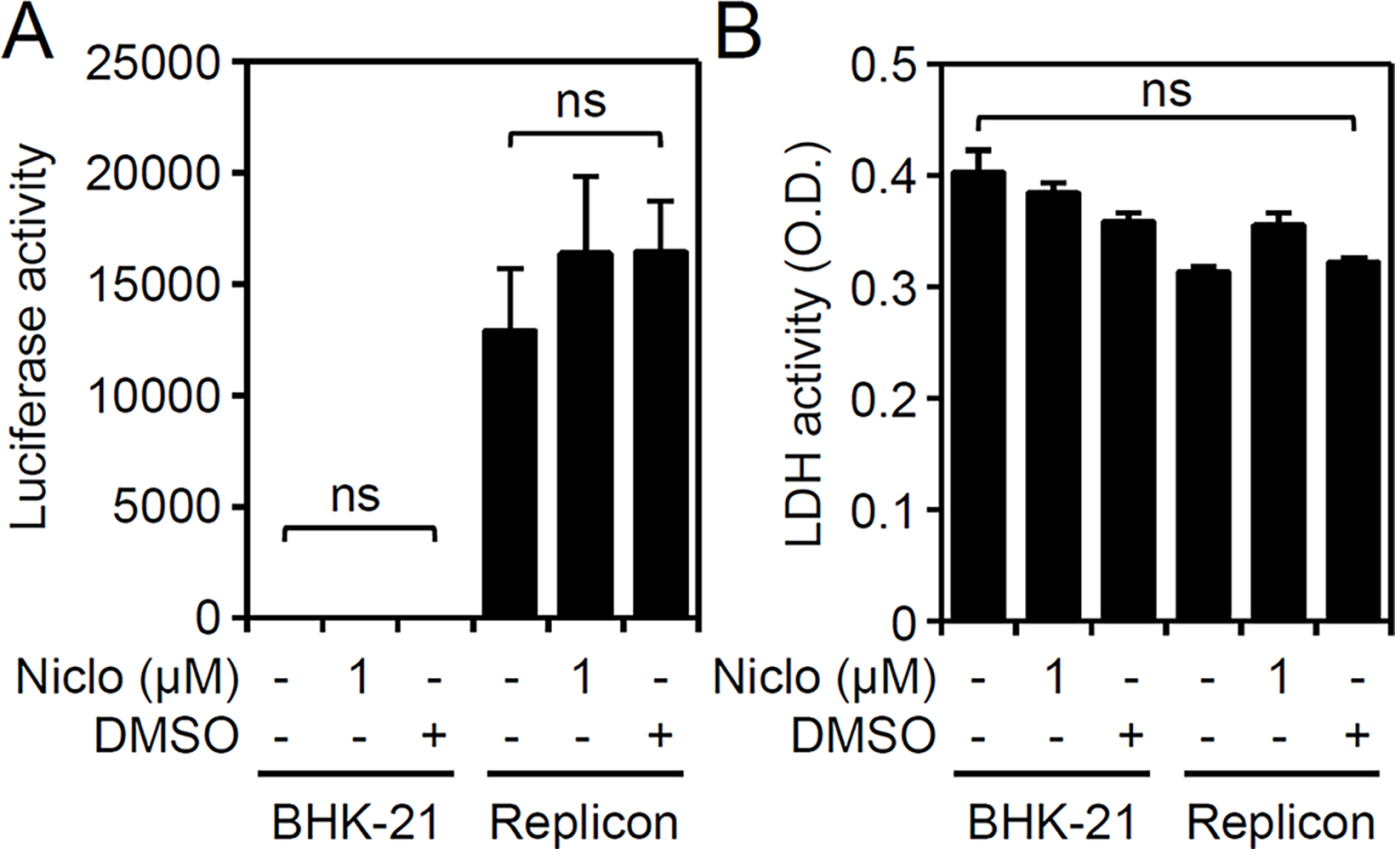
Niclosamide treatment does not repress firefly luciferase activity in BHK-D2-Fluc-SGR-Neo-1 cells. (A) Luciferase activity and (B) LDH assay in niclosamide (Niclo, 1 μM)-treated parental BHK-21 and BHK-D2-Fluc-SGR-Neo-1 cells (replicons) 24 h post-treatment. DMSO was used as a solvent control. Quantitative data are depicted as the mean ± SD of three independent experiments. ns, not significant.

We next examined other steps of the viral cell cycle by assessing firefly luciferase activity in BHK-D2-Fluc-SGR-Neo-1 cells, and we found that treatment with niclosamide caused neither direct inhibitory effects on replicon-based assay of viral genome translation or replication **(Fig 3A)** nor cytotoxicity in cells **(Fig 3B)**. These results indicate that blocking DENV infection with niclosamide had no direct inhibitory effects on FLuc activity in BHK-D2-Fluc-SGR-Neo-1 cells.

### Niclosamide initiated antiviral effects independent of inhibiting the mTOR, STAT3, and NK-kB signaling pathways

**Fig 4:**
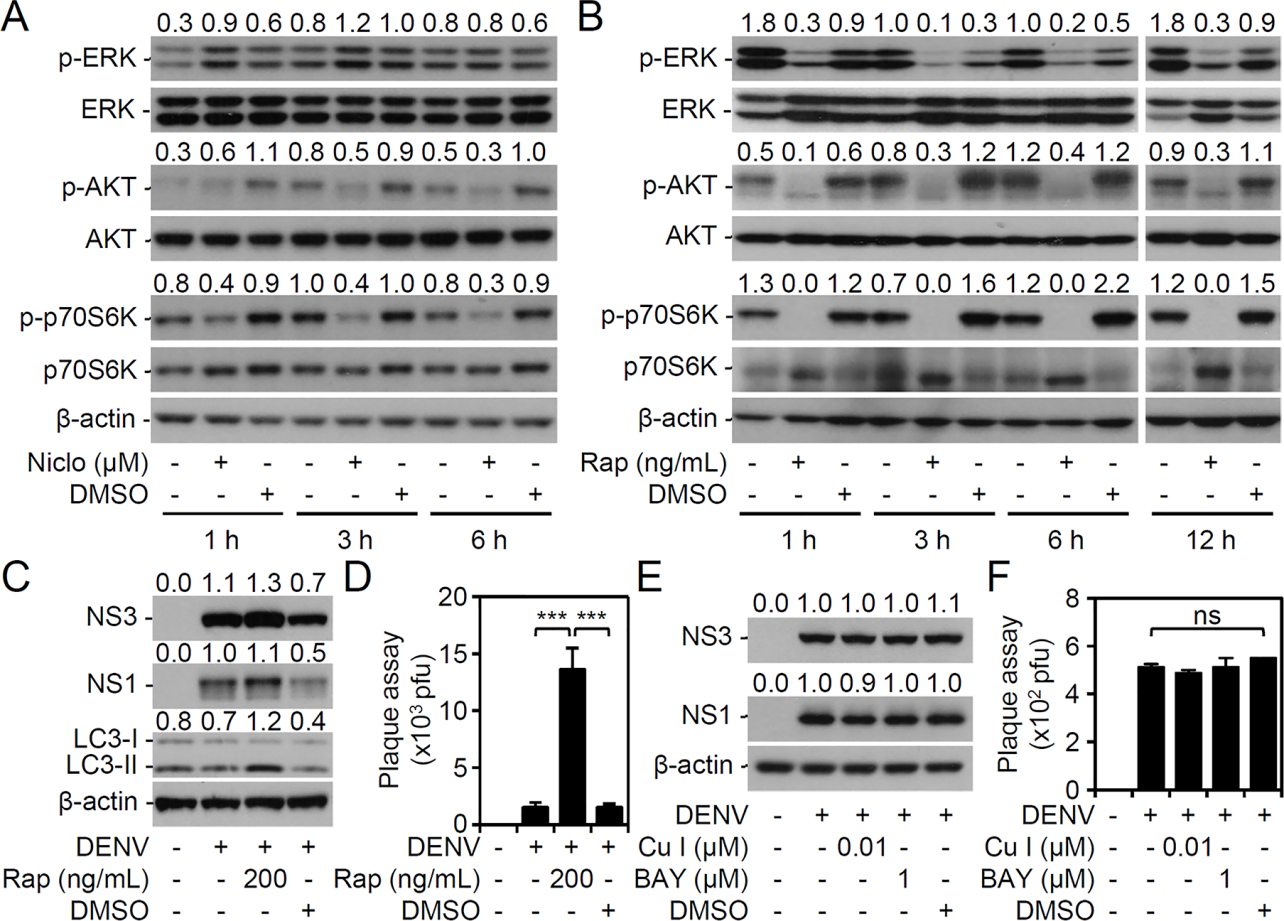
Niclosamide treatment reduces DENV infection independent of the mTOR, STAT3, and NF-κB signaling pathways. Representative Western blots showing the expression of the indicated proteins in (A) niclosamide (Niclo, 1 μM)- and (B) mTOR inhibitor rapamycin (200 ng/mL)-treated Neuro-2a cells for the indicated times. With or without rapamycin (Rap, 200 ng/mL), STAT3 inhibitor Cucurbitacin I (Cu I, 0.01 μM), and NF-κB inhibitor BAY 11–7082 (BAY, 1 μM) pretreatment, (C and E) representative Western blots showing the expression of the indicated proteins and (D and F) plaque assays showing viral release in DENV2 (MOI = 1)-infected Neuro-2a cells 48 h post-infection. DMSO was used as a solvent control. β-actin served as the internal control. The relative ratios of the measured proteins compared to those for total proteins and β-actin are also shown. The quantitative data are depicted as the mean ± SD of three independent experiments. *** *P <* 0.001. ns, not significant.

Niclosamide confers multiple therapeutic effects for the treatment of cancers, infections, and metabolic diseases by interfering activation of the mTOR, STAT3, and NF-κB signaling pathways [9, 10]. We next examined the effects of niclosamide on mTOR activation. A time-kinetic assay revealed the decreased phosphorylation of AKT and p70S6K in niclosamide-treated cells **(Fig 4A)**. These data indicate that treatment with niclosamide causes mTOR inhibition. Due to its action of abolishing the association of raptor (regulatory associated protein of mTOR) with mTOR, rapamycin is used as a classical mTORC1 inhibitor [23, 24]. Treatment with rapamycin effectively deactivated ERK, AKT, and p70S6K **(Fig 4B)**. We next evaluated the effects of mTOR inhibition on DENV infection in this study. Consistent with the previous studies in which rapamycin was reported to promote DENV infection through autophagy [25], rapamycin treatment enhanced the expression of viral proteins NS3 and NS1, induced autophagy with LC3II conversion **(Fig 4C)** and significantly (*P <* 0.001) facilitated viral release **(Fig 4D)**. Furthermore, treatment with STAT3 and NF-κB inhibitors blocked neither viral protein expression **(Fig 4E)** nor viral release **(Fig 4F)**, suggesting the independent roles of these possible targeting pathways. Taken together, these results indicate that niclosamide confers anti-mTOR activity, but the anti-dengue activity of niclosamide is mediated through a mTOR-independent pathway.

### Similar to other protonophores, niclosamide causes endosomal deacidification to suppress dsRNA replication and virus release

**Fig 5:**
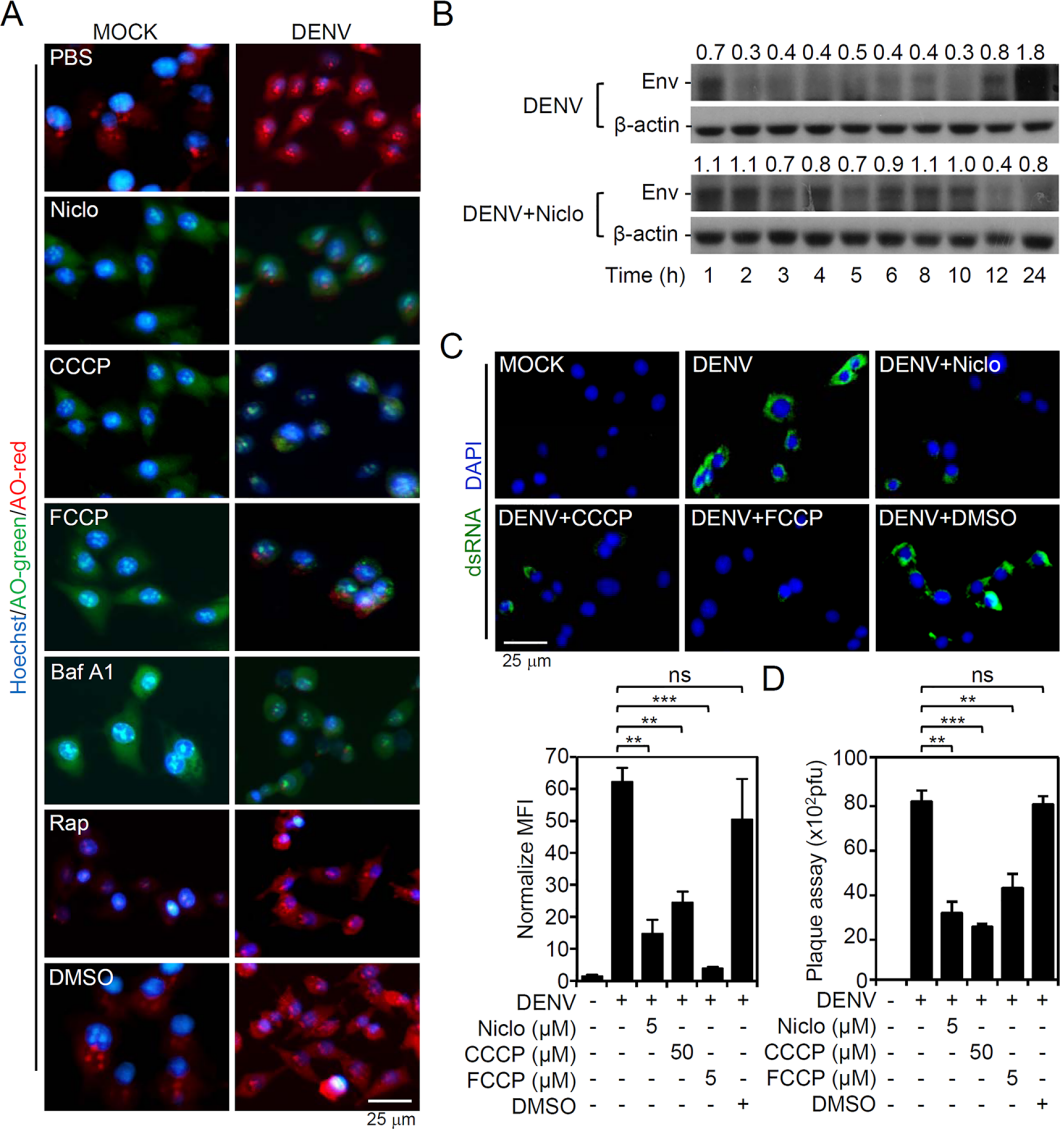
Niclosamide treatment causes endosomal deacidification to abolish DENV2 infection. BHK-21 cells were inoculated with DENV2 (MOI = 1) for 2 h in the presence of niclosamide (Niclo, 5 μM), the protonophores CCCP (50 μM) and FCCP (5 μM), the V-ATPase inhibitor (Baf A1, 100 nM) or rapamycin (Rap, 200 ng/mL). (A) Representative ratiometric live cell imaging of acridine orange (AO) staining showing acidic compartments (*red*). Nuclei were stained with Hoechst 33258 (*blue*). (B) Representative Western blot showing viral E protein expression in DENV2 (MOI = 1)-infected BHK-21 cells for the indicated times. The relative ratio to β-actin is shown. (C) Representative immunostaining and the relative mean fluorescence intensity (*MFI*) of viral dsRNA (*green*) at 6 h post-infection. (D) Plaque assay showing the level of viral replication 24 h post-infection. DMSO was used as a solvent control. For all images, representative data were selectively obtained from three individual experiments. Quantitative data are depicted as the mean ± SD of three independent experiments. * *P <* 0.05, ** *P <* 0.01, and *** *P <* 0.001. ns, not significant.

A structure-activity assay designated niclosamide as a protonophore which lowers the cytoplasmic pH to cause mTOR inactivation [26]. In endosomes, DENV requires a low-pH-dependent fusion for infectious genome entry into the cytoplasm. A pH-sensitive dye, AO, was used to examine whether selected drugs neutralize the low pH of endosomes during DENV infection. The results revealed that the low pH of endosomes (red) in DENV-infected cells was attenuated by treatment with niclosamide, the protonophores CCCP and FCCP, and the V-ATPase inhibitor bafilomycin A1 **(Fig 5A)**. Cells with niclosamide treatment are shown in green, suggesting that the pH was neutralized and endosome acidification was blocked. To assess the viral uncoating process, a time-kinetic expression of DENV E protein revealed that niclosamide interrupted E protein degradation during the early fusion stage of DENV infection **(Fig 5B)**. Furthermore, DENV dsRNA replication, as detected by immunostaining **(Fig 5C)**, and viral release, as determined by plaque assay **(Fig 5D)**, were significantly (*P <* 0.05) decreased by niclosamide, CCCP, and FCCP. These results reveal that niclosamide causes endosomal deacidification to inhibit dsRNA replication and viral release during DENV infection.

### Niclosamide treatment abolishes DENV infection *in vivo* and DENV-induced acute viral encephalitis-like disease progression

**Fig 6:**
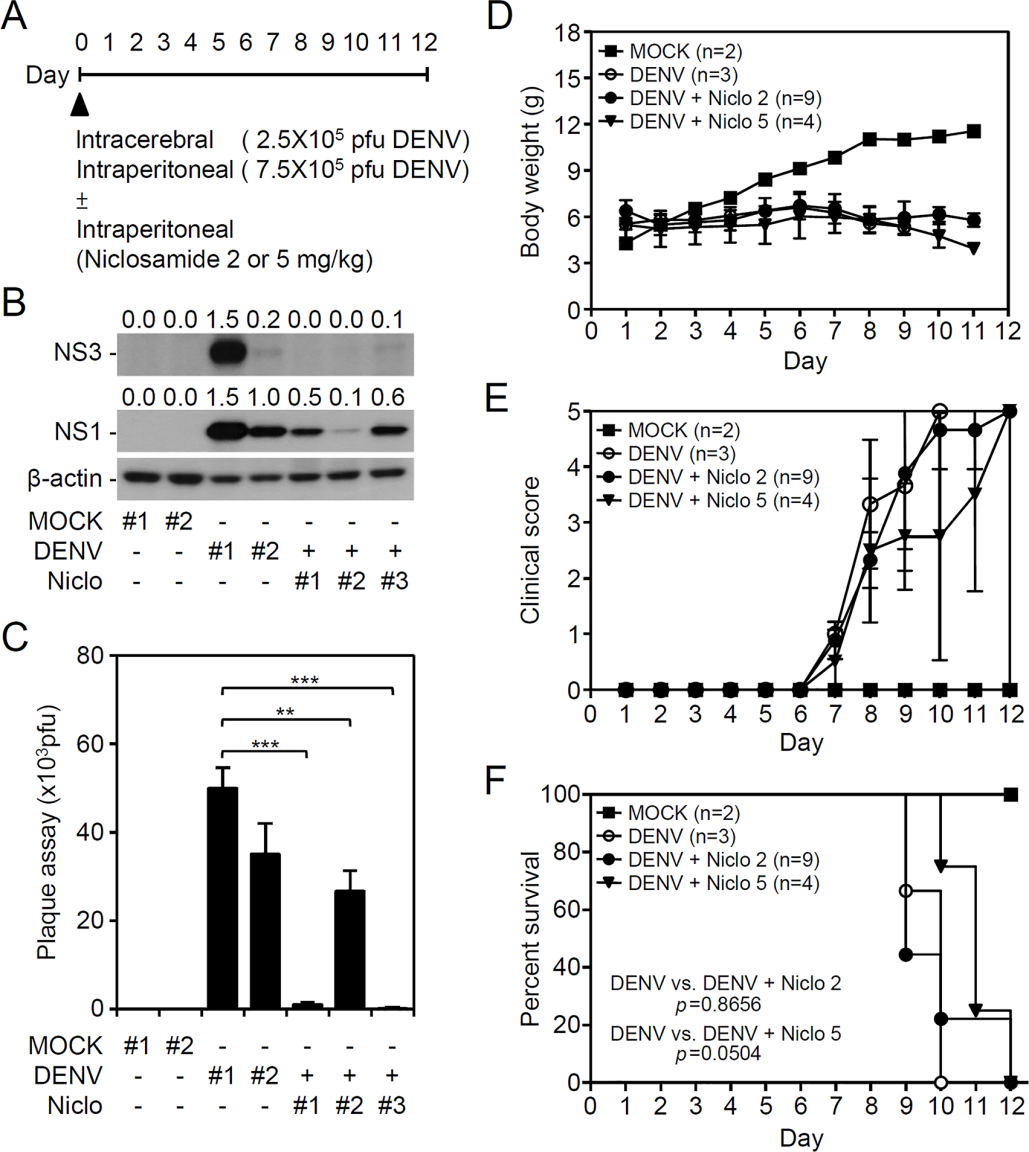
Niclosamide treatment reduces viral replication and increases the survival rate in suckling mice during DENV infection. (A) With or without niclosamide (Niclo; 2 mg/kg or 5 mg/kg) co-treatment, seven-day-old ICR suckling mice were inoculated with DENV2 by concurrent intracranial (2.5 × 10^5^ pfu) and intraperitoneal (7.5 × 10^5^ pfu) injections. (B) Western blot analysis of viral NS3 and NS1 protein expression. (C) Plaque assay of viral replication in the brains at 9 days post-infection. β-actin served as the internal control. The relative ratios of the measured proteins compared to β-actin are also shown. The quantitative data are depicted as the mean ± SD of three independent experiments. ** *P <* 0.01 and *** *P <* 0.001. Additionally, time-kinetic changes in (D) body weights, (E) clinical scores, and (F) survival rates were measured. *P* value is shown.

To further verify the antiviral effects of niclosamide against DENV infection *in vivo*, the viral replication, viral encephalitis, and mortality in DENV-infected ICR suckling mice were monitored accordingly [17, 20]. For this animal study, seven-day-old ICR suckling mice were inoculated with DENV2 by concurrent intracranial and intraperitoneal injections with or without niclosamide (2 or 5 mg/kg) co-treatment **(Fig 6A)**. According to the Western blot analysis of the NS3 and NS1 viral proteins **(Fig 6B)** and the plaque assays for detecting the production of infectious particles **(Fig 6C)**, DENV caused significant infection and replication in mouse brains at 9 days post-infection, and niclosamide inhibited viral protein expression and replication. We next monitored time-kinetic changes in clinical scores, which were graded according to the severity of illness as follows: 0 for healthy; 1 for minor illness, including weight loss, reduced mobility, and a hunchback body orientation; 2 for limbic seizures; 3 for moving with difficulty and anterior limb or posterior limb weakness; 4 for paralysis; and 5 for death, as previously described [17, 20]. First, DENV infection caused a dramatic loss in body weight in a time-dependent manner; however, niclosamide did not reverse these effects **(Fig 6D)**. A significant increase in clinical scores **(Fig 6E)** occurred in DENV-infected mice compared to mock-infected mice by 8 days post-infection. The survival rate of DENV-infected mice decreased by day 9 post-infection, and all of the mice died by day 10 post-infection **(Fig 6F)**. Co-treatment with niclosamide slightly reduced the DENV-induced disease progression and mortality. These data indicate that a single-dose treatment of niclosamide partly abolished encephalitic DENV infection in our model, which leads to neural impairment following viral replication.

## Discussion

According to our findings, treatment with the antiparasitic agent niclosamide confers antiviral activity, including effects on viral genome release, viral protein expression, dsRNA replication, and viral release *in vitro* in several DENV-infected cell lines. We also demonstrated that a single-dose administration of niclosamide partly reduces DENV replication *in vivo* as well as DENV-induced acute viral encephalitis-like symptoms, including progressive hunchback posture, limbic seizures, limbic weakness, paralysis, and lethality. These findings, along with the current study showing that niclosamide confers antiviral activity against replication of the flaviviruses Zika and DENV [7], we and others demonstrated the potential application of niclosamide treatment for inhibiting DENV infection *in vitro* and *in vivo*. In addition to its action against DENV, niclosamide has been demonstrated to be an antiviral agent against severe acute respiratory syndrome coronavirus [27], human rhinoviruses, influenza virus [19], Chikungunya virus [5], EBV [8], and Zika [6, 7, 28]. Repurposing niclosamide as an antiviral agent is therefore proposed.

In this examination of the antiviral action of niclosamide, inconsistent with the previous study [5] showing that niclosamide inhibits the entry of the Chikungunya virus into cells, our results showed a minor effect on the endocytosis of DENV in niclosamide-treated cells. The target of niclosamide for blocking viral entry/binding was not further addressed [5]. Furthermore, monitoring the antiviral IFN-β response in DENV-infected cells did not reveal immune enhancement by niclosamide stimulation. In contrast, niclosamide reduced IFN-β production, likely by suppressing DENV infection prior to the antiviral immune activation. Generally, niclosamide has been shown to block glucose uptake, oxidative phosphorylation, and anaerobic metabolism to kill tapeworm [9, 10]. Additionally, niclosamide can inhibit the Wnt/β-catenin, mTORC1, STAT3, NF-κB and Notch signaling pathways. For investigating niclosamide-induced antiviral actions, more validation is needed.

DENV replicon BHK-21 cells have been generated to assess the replication of the DENV genome [29]; however, niclosamide did not alter viral translation in DENV replicon cells in this study. Although the replicon cells contained the host and viral factors needed for viral genome replication, our findings revealed that the anti-DENV activity of niclosamide is independent of those factors. Currently, through the screening of 2,816 approved and investigational drugs, niclosamide has been identified as a potential viral inhibitor targeting the formation of the NS2B-NS3 protease of the flaviviruses Zika and DENV [7]. Considering that the maturation of DENV particles requires NS2B-NS3-mediated cleavage of the viral precursor polyprotein, the direct-acting antiviral agents, such as small-molecules, diaryl (thio)ethers, and cyclic peptides targeting the NS2B-NS3 protease are promising antiviral candidates [30–33]. As shown by Li *et al*. [7], three potent inhibitors of the NS2B-NS3 protease, including niclosamide, temoporfin, and nitazoxanide, have been confirmed to inhibit the complex formation of DENV NS2B and NS3. Moreover, these compounds have been shown to reduce the viral replication of DENV *in vitro* in human A549 cells. Given the niclosamide-based antiviral properties, targeting DENV NS2B-NS3, at least in part, could be implemented for reducing viral replication.

Niclosamide also exhibits multiple-targeted effects on cellular signaling pathways, such as mTOR, STAT3, and NF-κB [9, 10]. Inconsistent with niclosamide, inhibitors of STAT3 and NF-κB did not reduce DENV infection, but the mTOR inhibitor enhanced DENV replication. A recent study showed that niclosamide inhibits 12-O-tetradecanoylphorbol-13-acetate- and sodium butyrate-induced mTOR activation during EBV lytic replication [8]. Mechanistic studies indicate that niclosamide possesses protonophoric activity to dissipate protons from endosomes/lysosomes to the cytosol. The resulting increase in protons effectively lowers the cytoplasmic pH, causing mTOR inactivation [26]. However, inhibiting mTOR followed by autophagic induction facilitates DENV replication, likely by modulating lipid metabolism and promoting cell survival [12, 13]. In this study, in comparison with the direct-acting mTOR inhibitor rapamycin, niclosamide also inhibited the phosphorylation of AKT and p70S6K but induced LC3 conversion for autophagy. In contrast, rapamycin effectively enhanced DENV replication, indicating an enhanced role of autophagy in DENV infection. Although DENV promotes increased autophagy, niclosamide treatment should inhibit the infectious process of DENV prior to the induction of autophagy-facilitated DENV replication.

Endosomal acidification followed by viral RNA release is required for DENV replication [14, 15]. Targeting V-ATPase, a proton-pumping enzyme, inhibits the viral release from endosomes *in vitro* [16, 18, 34] and decreases DENV infection and neurotoxicity *in vivo* [17]. As reported by Jurgeit *et al*. [19], niclosamide acts as a proton carrier which blocks endosomal acidification. Here, we also provide evidence to confirm the inhibition of DENV-induced endosomal acidification, viral E protein degradation, dsRNA replication, and viral release by treatment with protonophores (niclosamide, CCCP, and FCCP). Retarding the DENV viral genome release by interfering with endosomal acidification could be another strategy utilizing the antiviral capability of niclosamide. Although Li *et al*. [7] showed that niclosamide treatment blocks the NS2B-NS3 complex formation *in vitro*, it is speculated that niclosamide-induced endosomal deacidification retards the early process of DENV infection rather than blocking NS2B-NS3.

Using our previous *in vivo* model of DENV infection showing that DENV causes replication in the brain followed by acute viral encephalitis-like symptoms in mice [17, 20], we showed that niclosamide not only reduces viral replication but also partly retards lethality in DEN-infected mice. In this study, to verify the antiviral activity of niclosamide, several DENV-infected cell lines *in vitro* and a mouse model of DENV infection *in vivo* were used. Further possible effects of niclosamide on the infectious processes were verified using the appropriate testing systems. It was found that niclosamide, similar to protonophores such as CCCP and FCCP, inhibits endosomal acidification to reduce viral genome release independent of inhibiting the mTOR, STAT3, and NF-κB signaling pathways and without effects on DENV endocytosis, antiviral IFN response, and viral translation. Co-administration of a single dose of niclosamide partly decreases DENV-induced acute viral encephalitis-like symptoms and mortality. A modified treatment with multiple doses is needed to validate its therapeutic efficacy. Although DENV replication may be blocked by niclosamide *in vitro* and *in vivo*, however, concurrent blocking pro-inflammatory and/or neurotoxic factors induced by DENV infection for neuroprotection is also needed for therapeutic consideration against dengue encephalitis. Furthermore, the blockade of endosomal acidification by niclosamide should be examined for its hazard effects on the synaptic activity in the neuronal cells although niclosamide causes endosomal deacidification independent of V-ATPase blockade [35]. In conclusion, together with the results of a recent study [7], our findings further highlight the repurposing application of niclosamide for antiviral drug development against DENV infection.

## Supporting information

S1 Fig Niclosamide treatment shows 50% cytotoxic concentration (CC50) in BHK-21 cells. LDH assay showing cytotoxicity in niclosamide (Niclo)-treated BHK-21 cells for 24 h with various concentrations. Treatment of Tween 20 served as the positive control. The relative percentages of cytotoxicity compared to Tween 20-induced 100% cytotoxicity are also shown. CC50 is indicated by dotted line.
(TIF)

S2 Fig Niclosamide treatment causes cytotoxicity in BHK-21 cells. Rhodamine 123-based staining followed by flow cytometry analysis showing mitochondrial membrane potential loss in niclosamide (Niclo)-treated BHK-21 cells for 12 h with various concentrations. The relative mean fluorescence intensity (MFI) are also shown. ns, not significant.
(TIF)

S3 Fig Niclosamide treatment shows a value of half maximal effective concentration (EC50) on the blockade of DENV infection in BHK-21 cells. Plaque assays showing viral release in DENV2 (MOI = 1)-infected BHK-21 (24 h) cells in the presence of niclosamide (Niclo). Virus particles are shown as the desired pfu amount for infection and as calculated as the percentage (%) of inhibition. The quantitative data are depicted as the mean ± SD of three independent experiments. ** *P <* 0.01 and *** *P <* 0.001. EC50 is indicated by dotted line.
(TIF)
