# Supplementary figures and images for: The antiparasitic drug niclosamide inhibits dengue virus infection by interfering with endosomal acidification independent of mTOR
Source: PLoS Negl Trop Dis. 2018 Aug 20;12(8):e0006715. doi: 10.1371/journal.pntd.0006715 (PMC6117097; doi:10.1371/journal.pntd.0006715)

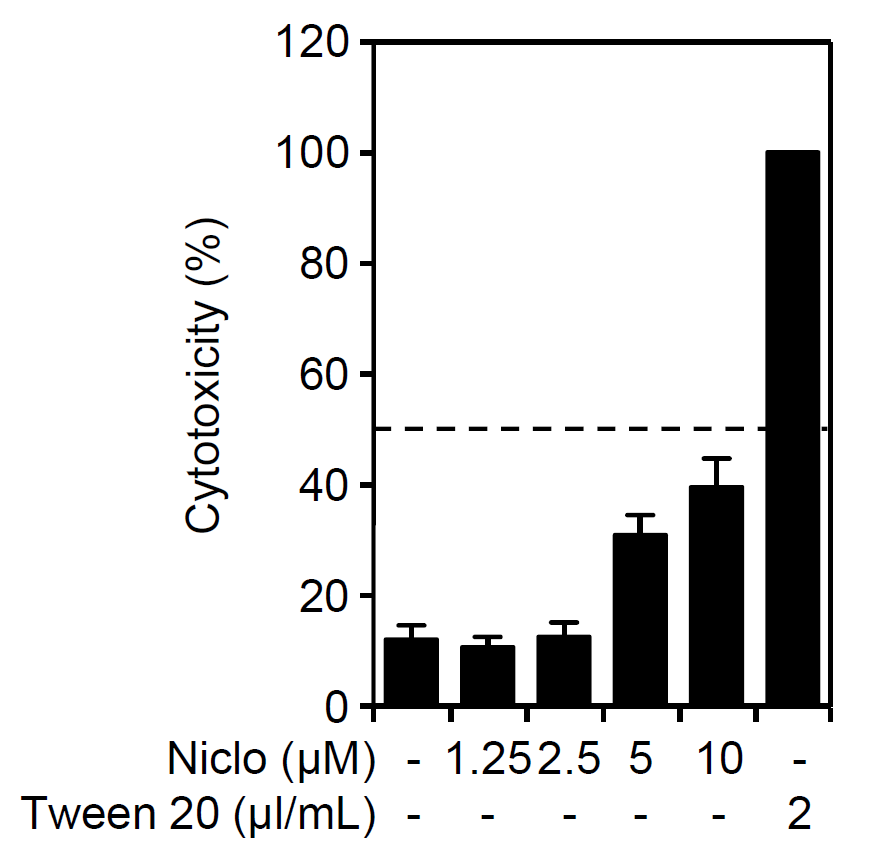

Supplement: S1 Fig — LDH assay showing cytotoxicity in niclosamide (Niclo)-treated BHK-21 cells for 24 h with various concentrations. Treatment of Tween 20 served as the positive control. The relative percentages of cytotoxicity compared to Tween 20-induced 100% cytotoxicity are also shown. CC50 is indicated by dotted line. (TIF) [file pntd.0006715.s001.tif]

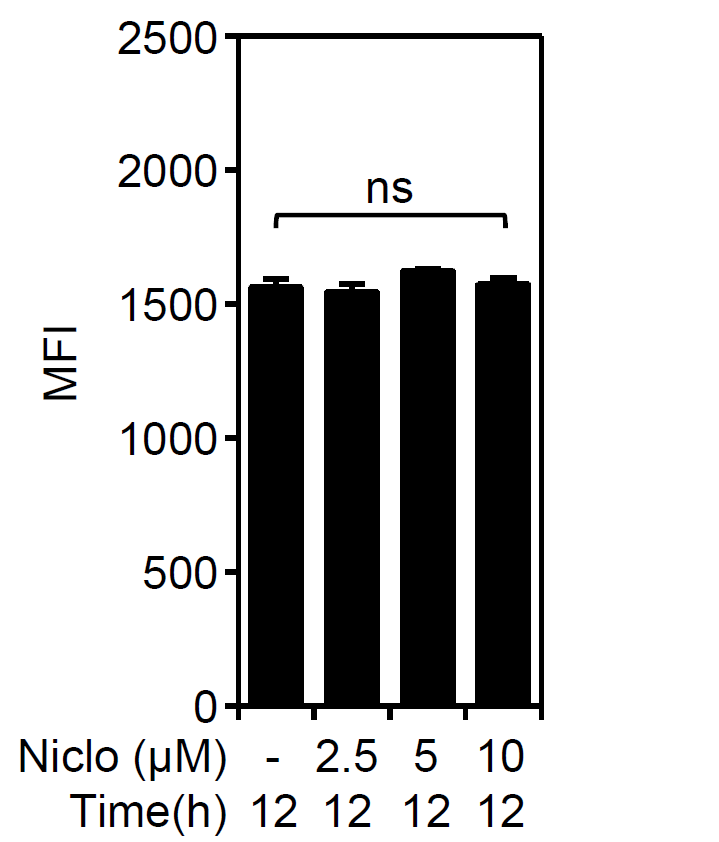

Supplement: S2 Fig — Rhodamine 123-based staining followed by flow cytometry analysis showing mitochondrial membrane potential loss in niclosamide (Niclo)-treated BHK-21 cells for 12 h with various concentrations. The relative mean fluorescence intensity (MFI) are also shown. ns, not significant. (TIF) [file pntd.0006715.s002.tif]

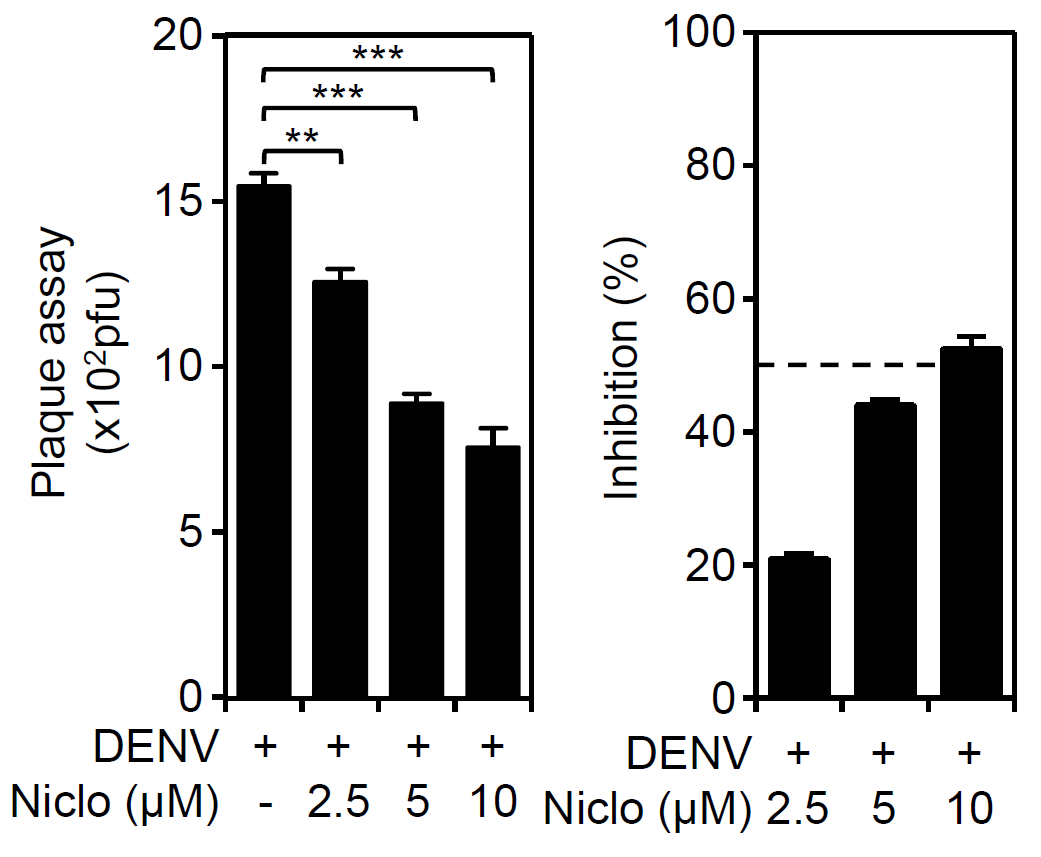

Supplement: S3 Fig — Plaque assays showing viral release in DENV2 (MOI = 1)-infected BHK-21 (24 h) cells in the presence of niclosamide (Niclo). Virus particles are shown as the desired pfu amount for infection and as calculated as the percentage (%) of inhibition. The quantitative data are depicted as the mean ± SD of three independent experiments. ** P < 0.01 and *** P < 0.001. EC50 is indicated by dotted line. (TIF) [file pntd.0006715.s003.tif]
